# Biometeorological insights into seasonal skin and coat adaptation in hairy goats from a semi-arid equatorial region

**DOI:** 10.1007/s00484-026-03194-5

**Published:** 2026-04-24

**Authors:** José Danrley Cavalcante dos Santos, Delfino Isac Belarmino Afo, Mikael Leal Cabral Menezes de Amorim, Tarsys Noan Silva Veríssimo, Geni Caetano Xavier Neta, Severino Guilherme Caetano Gonçalves dos Santos, Maria Isabelly Leite Maia, Luiz Arthur dos Anjos Lima, Larissa Kellen da Cunha Morais, Veerasamy Sejian, Ricardo Romão Guerra, Lucas Rannier Ribeiro Antonino Carvalho, Edilson Paes Saraiva

**Affiliations:** 1 Bioclimatology and Animal Ethology Group (BIOET), Federal University of Paraíba (UFPB), Areia, Paraíba Brazil; 2 Department of Veterinary Medicine, Faculty of Veterinary Medicine and Animal Science, Save University (UniSave), Chongoene,, Mozambique; 3 Modern Agricultural Consulting and Representations LTDA, São Paulo, Brazil; 4 Integrated Colleges of Patos (FIP), Paraiba, Brazil; 5 Paulista State University (UNESP), Jaboticabal, São Paulo, Brazil; 6 Department of Animal Science, State University of Maranhão (UEMA), São Luís, Brazil; 7 Paraíba Company for Research, Rural Extension and Land Regularization (EMPAER), Paraiba, Brazil; 8 Centre for Climate Resilient Animal Adaptation Studies, Animal Physiology Division, National Institute of Animal Nutrition and Physiology, Adugodi, Bangalore, 560030 Karnataka India; 9 Department of Veterinary Sciences, Federal University of Paraíba (UFPB), Areia, Paraíba Brazil; 10 Department of Physiology and Pharmacology, Karolinska Institutet, Stockholm, Sweden

**Keywords:** Skin characteristics, Thermal insulation, Tropical climate, Phenotypic acclimatization

## Abstract

The present study aimed to analyze annual variations in coat and skin characteristics of two goat breeds raised in an equatorial semi-arid environment. Samples were obtained from 50 multiparous does (25 Canindé and 25 Moxotó), evaluated quarterly over a one-year period. The following parameters were assessed: coat thickness (mm), hair density (hairs cm^−^²), and hair diameter and length (mm). Skin thickness was measured in micrometers (µm), as well as the area of sweat glands and cutaneous blood capillaries, expressed in µm cm^−^². The highest ambient temperatures and mean solar radiation were recorded between September and December. Significant differences (*P* < 0.05) were observed across periods for coat thickness, hair density, diameter, and length, as well as for the areas of cutaneous blood capillaries and sweat glands. In September, overall cutaneous thermal insulation in both breeds was reduced, which was associated with lower hair density and increased areas of blood capillaries and sweat glands (*P* < 0.05). It can be concluded that, under equatorial conditions, phenotypic adjustments in the morphological traits of the skin and coat contribute to the modulation of thermal insulation in goat breeds. Both Canindé and Moxotó goats adjusted insulation-related traits throughout the year. However, although both breeds exhibited marked phenotypic plasticity, Canindé goats showed greater variation in coat traits, suggesting distinct adaptive strategies in response to the environmental seasonality of the equatorial semi-arid region.

## Introduction

Small ruminant farming plays a strategic role in the agricultural economy of the Brazilian semi-arid region, particularly in the Northeast, where it holds economic, social, and cultural significance. Approximately 96% of goat production is concentrated in this area (Guabiraba et al. 2023), where the Canindé and Moxotó breeds are widely used in breeding systems due to their strong adaptive capacity developed through long-term natural selection in arid environments. These animals serve as an important source of high-quality protein in regions with limited agricultural productivity.

The climate in the Brazilian semiarid is characterized by high solar radiation, elevated air temperatures, and low annual precipitation (Silva et al. 2015). Among the main environmental variables affecting heat exchange in animals are air temperature and solar radiation (Maia et al. 2015; Silva et al. 2010). As endothermic animals, goats must maintain a balance between metabolic heat production, heat exchange with the environment through conduction, convection, and radiation, and heat dissipation via evaporative pathways (Fonseca et al. 2017; Maia et al. 2016; Silva and Maia 2013).

Key physical traits influencing overall thermal insulation include coat characteristics such as thickness, density, length, and fiber diameter (Maia et al.  2005a, 2005b) as well as skin features such as dermal thickness, hair bed density, sweat gland area and distribution (Jian et al. 2013; Costa et al. 2014).

Previous studies have identified seasonal variation and acclimatization in skin and coat thermal conductance in small ruminants from semiarid regions (Fonsêca et al. 2019; Façanha et al. 2020). For example, Amorim et al. (2019) observed seasonal variation in wool traits of Santa Inês and Morada Nova sheep around 7°S latitude, while Mascarenhas et al. (2023) examined skin and coat structure and sweating capacity in goats and sheep from the Brazilian semiarid.

Although previous studies have documented seasonal adaptations in other species and goat breeds, an important gap remains in understanding these mechanisms in Canindé and Moxotó goats raised in the Equatorial Semi-Arid region. In this context, two central research questions arise: (1) do Canindé and Moxotó goats exhibit seasonal variation in skin and hair coat morphology when raised under Equatorial Semi-Arid climatic conditions? and (2) if so, do these changes occur similarly between the two breeds? The results of this study are expected to advance the understanding of adaptive mechanisms and thermal tolerance in hairy goat breeds exposed to the challenging environmental conditions that characterize equatorial semi-arid regions.

## Materials and methods

### Research site, animals and experimental design

All animal-related procedures were evaluated and authorized by the Animal Use Ethics Committee of the Federal University of Paraíba (protocol no. 081/2015). Coat and skin samples were collected from 25 Moxotó goats and 25 Canindé goats on four specific dates: September 21, 2014, December 21, 2014, March 21, 2015, and June 21, 2015. In the experimental design, breed was considered the main plot, and season (sampling period) was treated as the subplot.

The animals were sourced from two locations: Moxotó goats from the Experimental Unit of the Federal University of Paraíba (07°23’27’’S, 458 m a.s.l.) and Canindé goats from a commercial farm (06°52’06’’S, 555 m a.s.l.). Both locations operated under a semi-intensive system, with grazing on native Caatinga vegetation and mineral supplementation. During the dry season, animals received additional feed. The average body condition score ranged from 3.0 to 3.5. Meteorological data including air temperature (°C), solar irradiance (W m⁻²), precipitation (mm), and mean day length were recorded between 2014 and 2015 at a weather station operated by the National Institute of Meteorology (07°29’20’’S, 436 m a.s.l.; Fig. 2).

### Coat characteristics

Samples were obtained from live animals according to the methodology described by Amorim et al. (2019). Measurements were taken from the area between the 12th and 13th thoracic vertebrae, approximately 10 cm below the spine, on the right side of each animal. The following coat traits were measured: coat thickness (mm), hair density (hairs cm⁻²), hair length (mm), and hair diameter (mm).

Coat thickness was measured using a digital caliper. Then, hair samples were collected from a 0.18 cm² area using specially adapted scissors and stored in paper envelopes. Hair density was calculated by counting the number of hairs within the sampled area and converting to hairs per cm². The length and diameter of the ten longest hairs per sample were measured using a digital caliper and a micrometer (Model IP40, Digimess, Brazil; accuracy ± 0.1 μm).

### Skin and Sweat Gland Characteristics

**Fig. 1:**
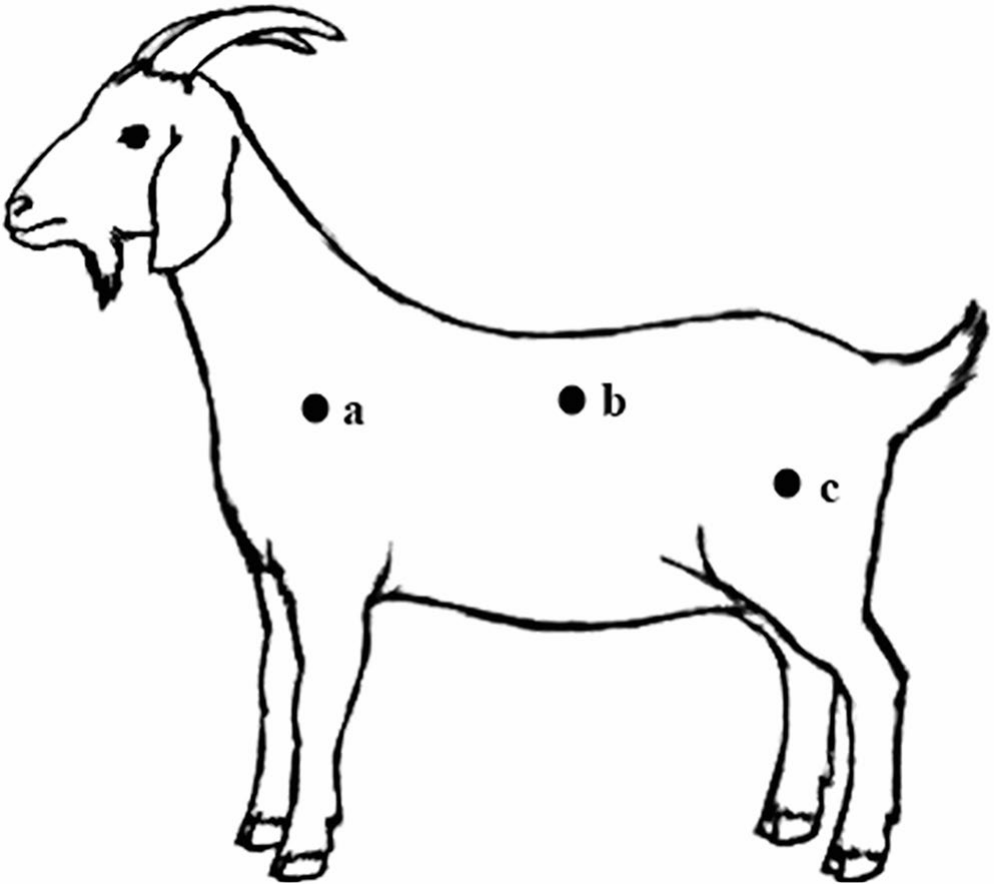
Regions of the body for skin sample collection (a = shoulder; b = side; c = leg)

Skin sampling and processing followed Amorim et al. (2019). Samples were collected from six goats of each breed using a 0.5 cm dermatological punch. Three body regions were sampled: (a) the upper central shoulder, (b) the lateral thorax between the 12th and 13th ribs (10 cm below the spine), and (c) the central leg region (10 cm below the ilium), as shown in Fig. 1. Sampling was performed 10 min after subcutaneous injection of 2 mL lidocaine hydrochloride (Lidovet - Bravet). Immediately after sample collection, the lesion site was treated with a topical ointment with healing and insect-repellent properties. Thereafter, the area was periodically treated with a repellent until complete healing.

Samples were fixed in 10% formalin and sent to a specialized laboratory for histological processing. Samples were embedded in paraffin, and 5 μm longitudinal sections were obtained using a microtome and stained with hematoxylin and eosin. Slides were scanned using a microscope (Olympus BX-60) equipped with a Motic digital camera and analyzed using Motic Images Plus 2.0 software.

The epidermal thickness was measured using 40× objectives, and dermal thickness using 5× objectives. Two photomicrographs of each lateral sample were captured, and four measurements per image were taken.

Sweat gland area was assessed using 5× objectives from two images per region (side, shoulder, leg), totaling 12 samples per region per season (6 animals × 2 images). Gland areas were calculated in µm² and converted to cm² of tissue.

Capillary area was measured using 40× objectives. Seven images were analyzed per region, resulting in 42 measurements per body region per season (6 animals × 7 images).

### Statistical analysis

Data were analyzed by analysis of variance (ANOVA), and means were compared using Tukey’s test at a 5% significance level. All analyses were performed using SAS (Statistical Analysis System) software. The following statistical models were used:

### Model 1

Yₖₗₘ = µ + R_i_ + Eₖ + RE_i_ₖ + RC_i_ₗ + e_i_ⱼₖₗₘ, for the analysis of hair coat surface characteristics, where:

- **Y**_**klm**_ is the j-th observation of hair coat surface traits;
- **µ** is the overall mean;
- **R**_**i**_ ​ is the fixed effect of breed (i = Canindé or Moxotó);
- **E**_**k**_ is the fixed effect of season (k = September, December, March, June);
- **RE**_**ik**_ is the breed × season interaction; and.
- **e**_**ijklm**_ is the random residual error.

### Model 2

Yₖₗₘ = µ + R_i_ + Eₖ + RE_i_ₖ + RC_i_ₗ + e_i_ⱼₖₗₘ, for the analysis of skin morphological characteristics, where:

- **Y**_**klm**_ is the m-th observation of skin traits;
- **µ** is the overall mean;
- **R**_**i**_ is the fixed effect of breed;
- **E**_**k**_ is the fixed effect of season;
- **RE**_**ik**_ is the breed × season interaction;
- **RC**_**il​**_ is the breed × body region interaction; and.
- **e**_**ijklm**_ is the random residual error.

These models were fitted to evaluate the main effects and their interactions on coat and skin morphological traits.

## Results

### Meteorological variables

**Fig. 2:**
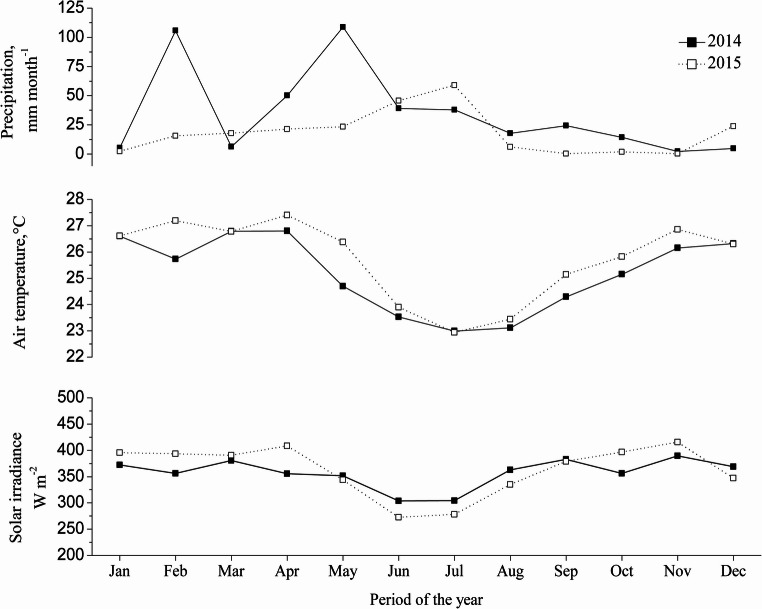
Monthly pattern for climate variables

Meteorological data from 2014 to 2015 (Fig. 2) revealed a well-defined seasonal pattern, characterized by lower air temperatures and reduced solar irradiance from June to September. From September to December, in both years, mean air temperature and solar irradiance increased progressively, and this period coincided with the lowest precipitation levels.

### Features of the surface of the coat

**Fig. 3:**
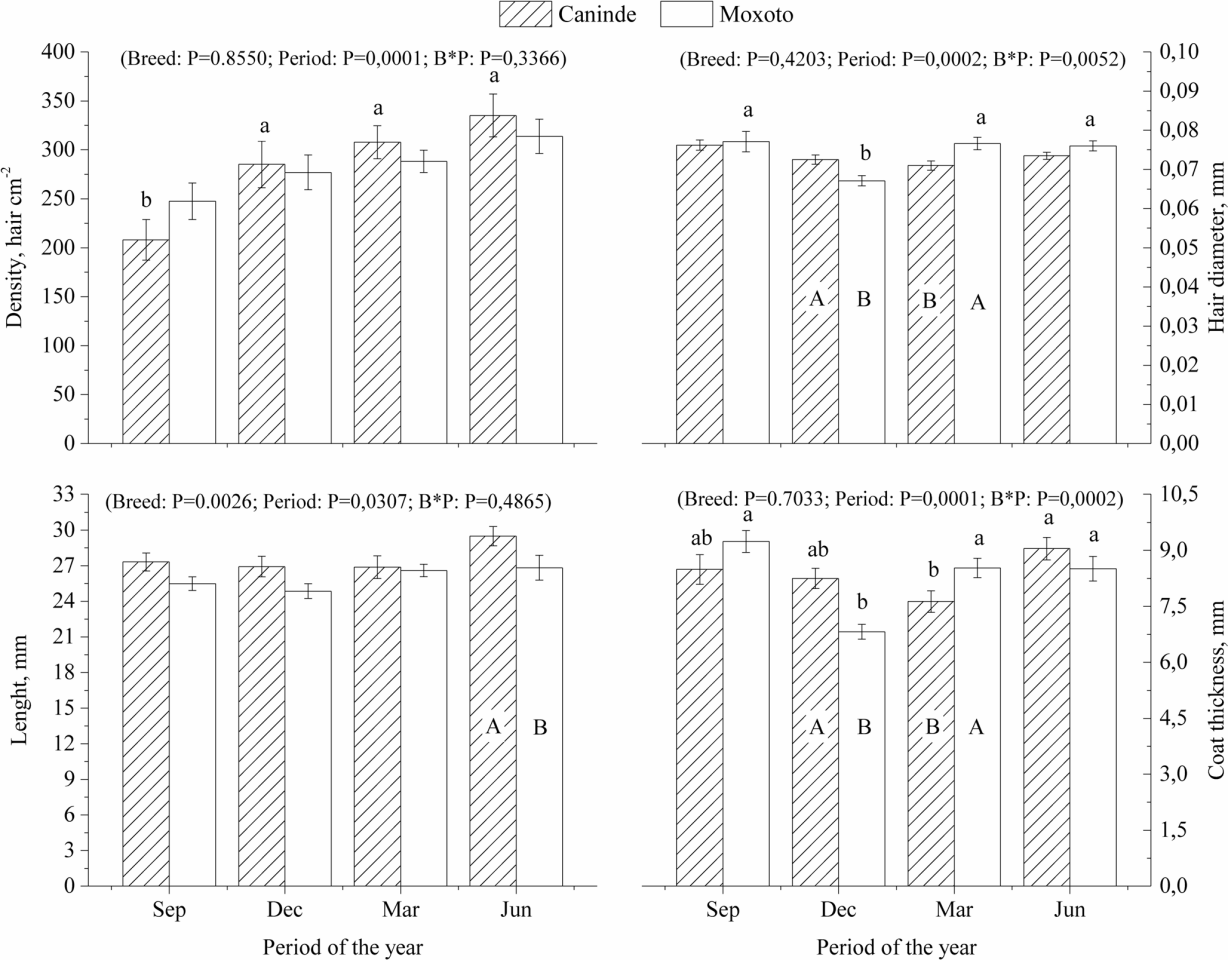
Annual pattern of coat characteristics of Canindé and Moxotó goats raised in the equatorial semiarid region. Capital letters correspond to the effect of the period of the year, and lowercase letters correspond to the effect of the breed. Sep = September 2014; Dec = December 2014; Mar = March 2015; Jun = June 2015; B*P = interaction between fixed factors (breed and season of the year). Data are presented as mean ± SEM

The goats of the Canindé breed are predominantly dark-colored, while the Moxotó breed is white-colored. The annual pattern of coat characteristics of Canindé and Moxotó goats is presented in Fig. 3. With the exception of coat density and length, fixed effects analyses (breed vs. season) demonstrated a significant interaction (*P* < 0.05) for coat diameter and thickness.

In June, the coat of Canindé goats was significantly longer (*P* < 0.05) than that of Moxotó, while both breeds showed reduced coat density in September. In December, hair diameter was significantly smaller (*P* < 0.05) for both breeds. In addition, greater coat diameters and thickness (*P* < 0.05) were observed in December for Canindé goats and in March for Moxotó goats (Fig. 3).

### Morphological characteristics of the skin

**Fig. 4:**
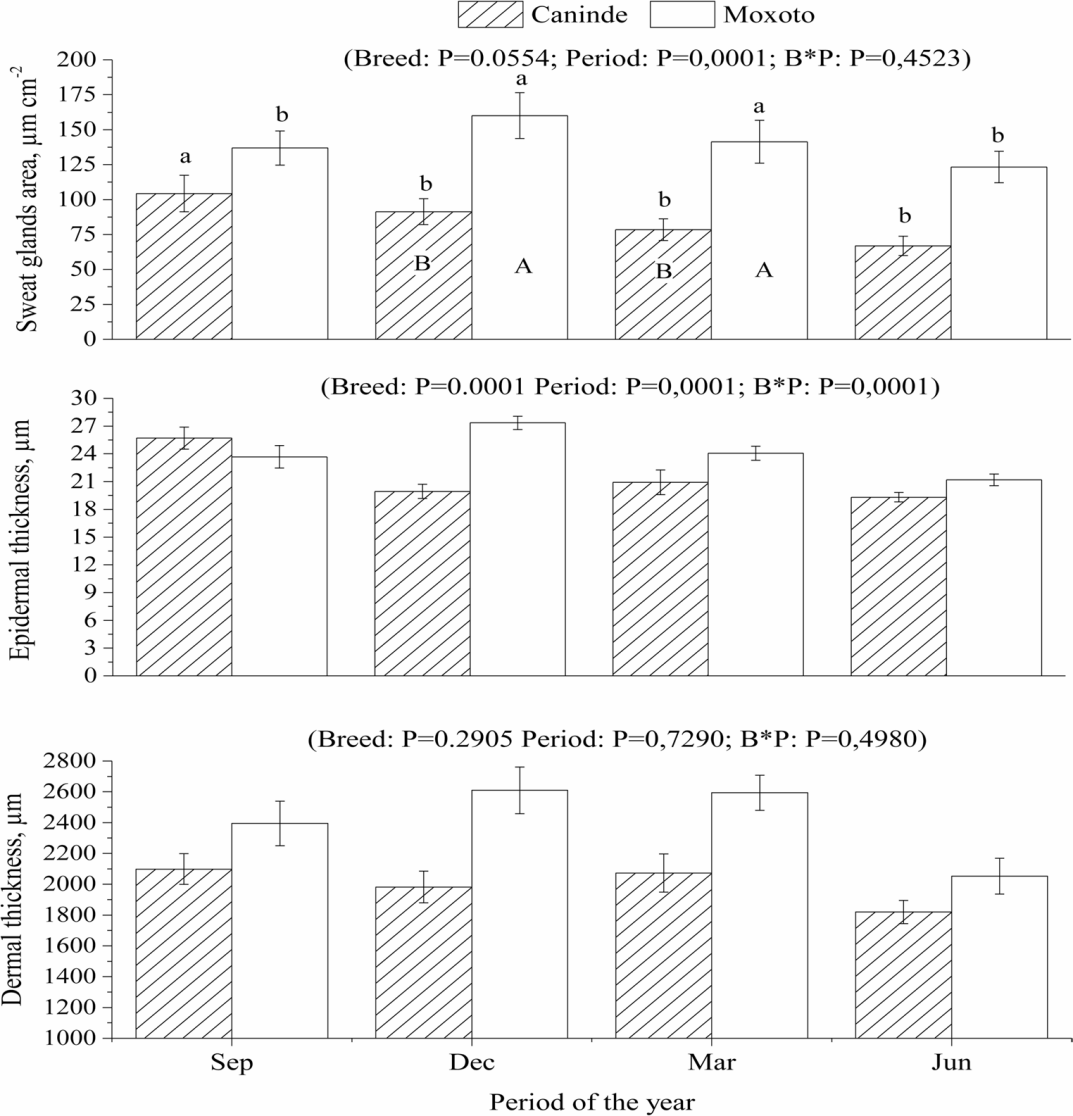
Annual pattern of sweat gland area, epidermal and dermal thickness of Canindé and Moxotó goats raised in the equatorial semiarid region. Uppercase letters correspond to the effect of the period of the year, and lowercase letters correspond to the effect of the breed. Sep = September 2014; Dec = December 2014; Mar = March 2015; Jun = June 2015; B*P = Interaction between fixed factors (breed and time of year). Data are presented as mean ± SEM

The annual variation in dermal and epidermal thickness, as well as in the area of sweat glands and cutaneous blood capillaries in Canindé and Moxotó goats, is shown in Figs. 3 and 4.

A significant interaction between the fixed effects of breed and period of the year was observed for epidermal thickness and cutaneous capillary area (*P* < 0.05), indicating breed-specific responses throughout the annual cycle. In contrast, dermal thickness was not influenced by the interaction between these factors, and no significant differences were detected between breeds throughout the year (*P* > 0.05; Fig. 4).

Specifically, in December, March, and June, Moxotó goats exhibited greater epidermal thickness (*P* < 0.05) than Canindé goats (Fig. 3). In September, however, Canindé goats showed greater epidermal thickness (*P* < 0.05) than Moxotó goats, revealing a seasonal reversal in the pattern observed between breeds.

**Fig. 5:**
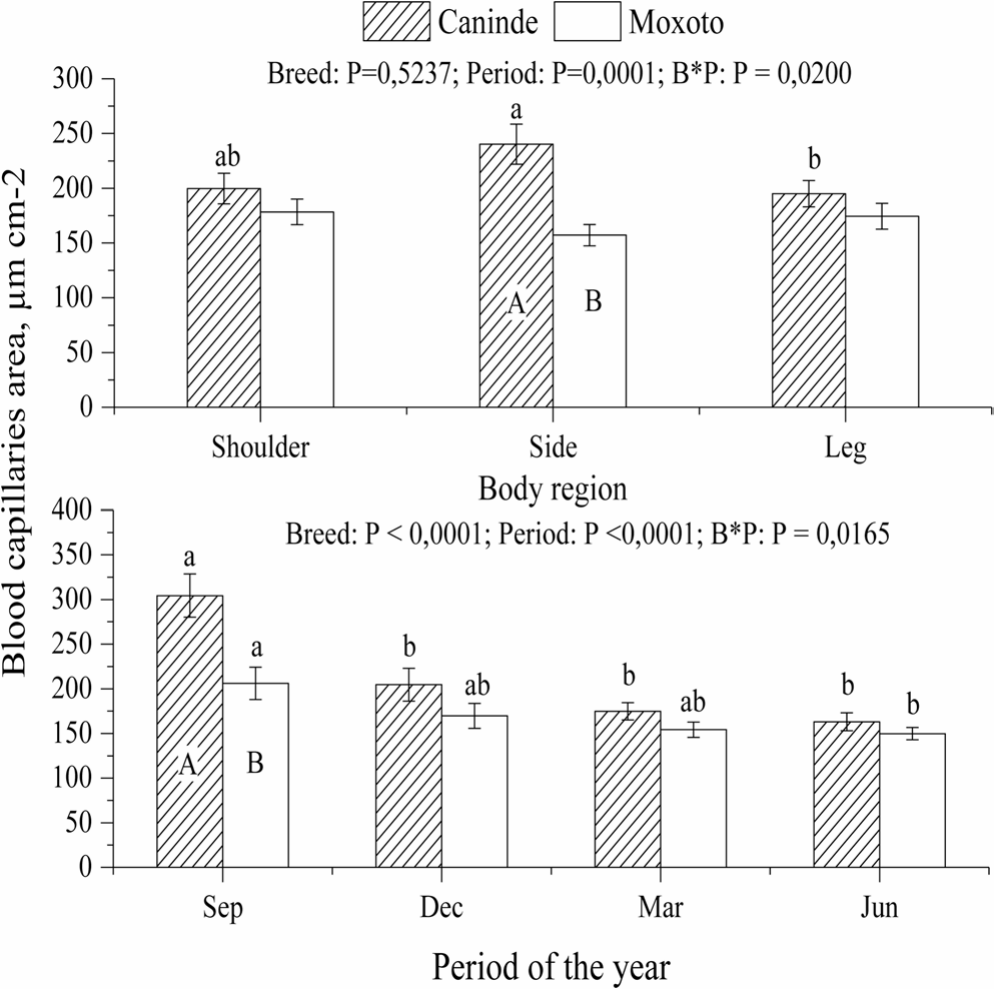
Differences in annual pattern and body region for blood capillary area of ​​Canindé and Moxoto goats raised in the equatorial semiarid region. Uppercase letters correspond to the effect of the period of the year, and lowercase letters correspond to the effect of the breed. Sep = September 2014; Dec = December 2014; Mar = March 2015; Jun = June 2015; B*P = Interaction between fixed factors (breed and season of the year). Data are presented as mean ± SEM

Moxotó goats had a larger sweat gland area (*P* < 0.05) than Canindé goats throughout the year (Fig. 4). Sweat gland area did not show significant differences (*P* > 0.05) between the shoulder, side and leg regions for both breeds. In contrast, the interaction between the fixed effects (breed × body region) for blood capillary area was statistically significant (*P* < 0.05), with the largest blood capillary area observed in Canindé goats as shown in Fig. 5.

## Discussion

### Meteorological variables

In regions characterized by consistently high temperatures, such as the semi-arid zone, thermal environmental conditions strongly influence the morphological traits of animals. In this specific geographic context, the solar tilt angle results in a relatively stable pattern of solar irradiance, air temperature, and photoperiod throughout the year (Silva 2006).

Indeed, climatic fluctuations in equatorial regions are generally minimal, as evidenced by the meteorological data collected during this study from 2014 to 2015. It is noteworthy that in both years, increases in solar irradiance, air temperature, and day length began in September, marking a seasonal transition despite the equatorial latitude.

These environmental factors act as zeitgebers external cues that synchronize biological rhythms triggering physiological adjustments in goats, including modifications in coat and skin characteristics over time. Such adjustments, known as phenotypic acclimation, are part of the animal’s strategy for enhancing thermal adaptation to the environment (Mascarenhas et al. 2023).

### Features of the surface of the coat

Coat color can influence the adaptive capacity of goats raised in semi-arid regions. In this context, since the two breeds analyzed in this study have distinct coat colors, they may exhibit different strategies of thermal adaptation or rely on alternative morphological mechanisms. The physical properties of coat pigmentation influence the amount of solar radiation absorbed by the body surface and, consequently, the surface temperature (Aleena et al. 2020; Laouadi et al. 2020). Black-coated goats exposed to high solar radiation exhibit higher surface temperatures than white-coated goats, suggesting greater susceptibility to thermal stress under direct sunlight (Maia et al. 2015).

However, higher surface temperatures in black goats should not necessarily be interpreted as a thermal disadvantage. In fact, in black coats, most of the solar radiation is absorbed near the outer layer of the fur, whereas in white coats, radiation penetrates deeper, depositing heat closer to the skin (Gebremedhin et al. 1997; Jiang et al. 2005). This difference in depth of heat absorption may result in distinct internal thermal dynamics between animals of different coat colors.

When facing thermal challenges, homeothermic animals activate thermoregulatory responses and undergo morphological adjustments to improve their adaptive performance in semi-arid environments (Silveira et al. 2024). As shown in Fig. 3, the longer coat observed in Canindé goats during June likely contributes to increased thermal insulation, favoring heat retention. This is particularly relevant in this period, when nighttime temperatures can drop below the animals’ core temperature. Ribeiro et al. (2017) reported that variations in hair traits help maintain homeostasis, and seasonal modulation of coat density contributes to the stability of rectal temperature, improving animal welfare across seasons.

The lower coat density observed in both breeds in September is considered beneficial in hot climates, as it enhances air circulation within the coat, facilitating the dissipation of trapped warm air. This promotes heat loss via forced convection generated by wind. Animals with thinner coats, such as the breeds studied, may avoid overheating during peak heat by allowing efficient daytime heat dissipation, while storing body heat for gradual release at night (Fonseca et al. 2016). However, under cooler night conditions, these animals may rely on huddling behavior to reduce heat loss due to limited insulation (Fonseca et al. 2019). This adaptive pattern contrasts with that observed in goats raised in temperate environments, such as the Palmera breed, which consistently exhibit a thicker and denser coat with longer hairs. These traits constitute an adaptive advantage during cold periods, as they enhance thermal insulation and reduce heat loss (Silva et al. 2025).

The greater coat thickness and hair diameter (*P* < 0.05) observed in December for Canindé goats, and in March for Moxotó goats, reflects seasonal morphological plasticity. Among the two breeds, Canindé goats exhibited the largest hair diameter in December, likely due to their black coat, which absorbs more radiation and increases surface heating. In contrast, Moxotó goats reflect more solar radiation, reducing the risk of heat accumulation. Therefore, larger hair diameter may enhance thermal conductivity via molecular conduction, which can be advantageous in hot environments (Ribeiro et al. 2017).

### Morphological characteristics of the skin

Dermal thickness did not show significant variation (*P* > 0.05) between breeds throughout the year, and the epidermis plays a crucial role in protection against solar radiation. The greater epidermal thickness observed in Moxotó goats (*P* < 0.05) compared to Canindé in the months of December, March, and June (Fig. 4) may reflect enhanced protection capacity. In contrast, non-reflected radiation is transmitted more rapidly and penetrates deeper into the epidermal layers (Façanha et al. 2010). Therefore, these animals need to adjust to avoid excessive heat accumulation and prevent possible internal damage caused by radiation. In temperate regions, darker coat coloration may favor solar radiation absorption during cold periods, thereby contributing to greater body heat gain and improved thermoregulation.

The larger area of ​​sweat glands in Moxotó goats (*P* < 0.05) throughout the year (Fig. 4) may be associated with the tendency of these goats with light coats to lose more heat through cutaneous evaporation compared to those with black coats. Mascarenhas et al. (2023) reported that goats have thinner epidermis, lighter hair and lower density, hence a higher density of sweat glands per area, which may facilitate the loss of sensible and latent heat. This characteristic is also associated with greater greater sensitivity of these goats to ultraviolet radiation and the need to dissipate heat, resulting in greater sweating and, consequently, an increase in the area of ​​active sweat glands. In contrast, goat breeds originating from temperate regions exhibit lower sweat gland densities, reinforcing the influence of hot and dry environments on the expression of these morphological traits (Costa et al. 2014).

The area of the dermis occupied by sweat glands did not show statistically significant differences (*P* > 0.05) between the shoulder, lateral, and leg regions, suggesting that the size and distribution of these glands are uniform in these areas. Similarly, Aiura et al. (2014) and Mascarenhas et al. (2023) found no variations in the gland area between the cervical, thoracic, and gluteal regions in Saanen goats and Santa Inês sheep. In the same study, Mascarenhas et al. (2023) observed that although Moxotó goats, which exhibited fewer sweat glands in the thoracic and gluteal regions compared to the cervical region. However, there were no differences in the volume of sweat excreted, demonstrating that sweat excretion is not homogeneous throughout the body and that the number of sweat glands does not influence the volume of sweat excreted, but rather its functionality (Medeiros et al. 2015).

The greater blood capillary area observed in Canindé goats (Fig. 5) may be associated with a higher demand for heat dissipation, possibly resulting from greater solar radiation absorption and the consequent increase in body temperature. This enlarged capillary area appears to reflect enhanced vasodilation and increased peripheral blood circulation, favoring heat transfer from deeper tissues to the skin surface. In addition, this process may stimulate sweat gland activity, thereby enhancing evaporative heat loss. As reported by Alvarez et al. (1970) and Dellmann and Brown (1982), elevated temperatures increase blood supply to the epidermis and intensify glandular activity.

This adjustment is particularly important in goats raised in semi-arid regions, where ambient temperature frequently approaches body temperature. Under such conditions, the reduced thermal gradient between the animal and the environment limits sensible heat dissipation and may compromise thermal homeostasis and welfare. In this context, increased cutaneous vascularization represents an important adaptive mechanism, as it enhances peripheral heat dissipation when non-evaporative pathways become less efficient.

These adaptations directly affect thermoregulatory efficiency, productive performance, and the sustainability of low-input extensive production systems in the Equatorial Semi-Arid region. They may contribute to greater hardiness, improved use of feed resources during critical periods, and enhanced production stability. Nevertheless, seasonal environmental variation may require complementary management strategies, such as the provision of additional shade during hotter periods, in order to optimize welfare and productivity. Furthermore, these morphological traits should be considered in breeding programs, with emphasis on the selection of genotypes adapted to local climatic conditions. Thus, skin and coat morphological traits not only reflect essential adaptive mechanisms for survival, but also have relevant zootechnical and economic implications, strengthening the resilience of goat production systems in semi-arid environments.

## Conclusion

Canindé and Moxotó goats exhibit marked phenotypic plasticity, expressed through seasonal adjustments in important skin and coat morphological traits that favor the maintenance of thermal homeostasis under Equatorial Semi-Arid conditions. These findings deepen the understanding of the adaptive biology of native breeds and highlight their value as strategic genetic resources for the sustainability of goat production systems in climatically challenging environments. Future studies should focus on elucidating the genetic basis of these traits in order to support breeding programs aimed at enhancing climate resilience and productive efficiency.

## Data Availability

Not applicable.
